# Targeting Glutamine Metabolism to Enhance Immunoprevention of EGFR‐Driven Lung Cancer

**DOI:** 10.1002/advs.202105885

**Published:** 2022-07-21

**Authors:** Mofei Huang, Donghai Xiong, Jing Pan, Qi Zhang, Shizuko Sei, Robert H. Shoemaker, Ronald A. Lubet, Luis M. Montuenga, Yian Wang, Barbara S. Slusher, Ming You

**Affiliations:** ^1^ Center for Cancer Prevention Houston Methodist Cancer Center Houston Methodist Research Institute Houston TX 77030 USA; ^2^ Chemopreventive Agent Development Research Group Division of Cancer Prevention National Cancer Institute Bethesda MD 20850 USA; ^3^ Program in Solid Tumors and Biomarkers Center for Applied Medical Research (CIMA) University of Navarra Pamplona 31009 Spain; ^4^ Department of Histology and Pathology University of Navarra Pamplona 31009 Spain; ^5^ Respiratory Tract Tumors Group Idisna Pamplona 31000 Spain; ^6^ Respiratory Tract Tumors Program CIBERONC Madrid 28013 Spain; ^7^ Johns Hopkins Drug Discovery Johns Hopkins University School of Medicine Baltimore MD 21205 USA; ^8^ Department of Neurology Johns Hopkins University School of Medicine Baltimore MD 2128 USA

**Keywords:** epidermal growth factor receptor vaccines, glutamine metabolism, JHU‐083, lung tumorigenesis, tumor immune microenvironment

## Abstract

Lung cancer is the leading cause of cancer death worldwide. Vaccination against EGFR can be one of the venues to prevent lung cancer. Blocking glutamine metabolism has been shown to improve anticancer immunity. Here, the authors report that JHU083, an orally active glutamine antagonist prodrug designed to be preferentially activated in the tumor microenvironment, has potent anticancer effects on EGFR‐driven mouse lung tumorigenesis. Lung tumor development is significantly suppressed when treatment with JHU083 is combined with an EGFR peptide vaccine (EVax) than either single treatment. Flow cytometry and single‐cell RNA sequencing of the lung tumors reveal that JHU083 increases CD8^+^ T cell and CD4^+^ Th1 cell infiltration, while EVax elicits robust Th1 cell‐mediated immune responses and protects mice against EGFR^L858R^ mutation‐driven lung tumorigenesis. JHU083 treatment decreases immune suppressive cells, including both monocytic‐ and granulocytic‐myeloid‐derived suppressor cells, regulatory T cells, and pro‐tumor CD4^+^ Th17 cells in mouse models. Interestingly, Th1 cells are found to robustly upregulate oxidative metabolism and adopt a highly activated and memory‐like phenotype upon glutamine inhibition. These results suggest that JHU083 is highly effective against EGFR‐driven lung tumorigenesis and promotes an adaptive T cell‐mediated tumor‐specific immune response that enhances the efficacy of EVax.

## Graphical abstract

Glutamine blockade with JHU083 enhances the efficacy of an epidermal growth factor receptor (EGFR) peptide vaccine in controlling the development and growth of EGFR‐driven lung cancer. JHU083 increases infiltration of anti‐tumor CD8^+^ T cells and Th1 T cells and decreases immune suppressive myeloid‐derived suppressor cells, regulatory T cells, and CD4^+^ Th17 cells in EGFR‐mutated lung tumors.

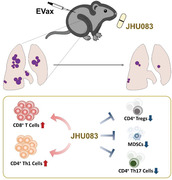

## 1 Introduction

Tumor cells have unique metabolic features to support their anabolic needs in a nutrient‐deficient microenvironment.^[^ ^1^ ^]^ Similar to their high demand for glucose, tumor cells also heavily rely on glutamine as the fuel to fulfill bioenergetic requirements. Glutamine provides anaplerotic nitrogen and a carbon source for many macromolecule syntheses to support cancer cell growth.^[^ ^2^ ^]^ Some oncogenes also rewire tumor cells with distinct glutamine consumption phenotypes.^[^ ^3^ ^]^ For example, lung cancer with epidermal growth factor receptor (EGFR)‐activating mutations exhibit elevated glutamine metabolism.^[^ ^4^ ^]^ Meanwhile, glutamine is an essential nutrient for immune cell proliferation and function within the tumor microenvironment (TME).^[^ ^5^ ^]^ The rapid consumption of glutamine by tumor cells leads to a TME that is restricted in the amount of glutamine for immune cells. Therefore, tumor cell glutamine utilization is potentially a metabolic checkpoint that inhibits immune cell‐mediated anti‐tumor responses.

Recently, scientists developed a novel agent, JHU083, to inhibit glutamine metabolism in cancer cells.^[^ ^6^ ^]^ JHU083 is a prodrug of 6‐diazo‐5‐oxo‐norleucine (DON), a broad antagonist for glutamine utilizing enzymes that has shown striking anticancer effects. In clinical trials, however, unacceptable gastrointestinal tract‐related toxicities were observed.^[^ ^7^ ^]^ Recently, a series of DON prodrugs, including JHU083, were designed to circulate in an intact and inert form in plasma but be preferentially activated in the TME by tumor‐enriched enzymes (e.g., esterases and peptidases) that cleave the pro‐moieties.^[^ ^8^ ^]^ This prodrug strategy has been shown to deliver DON preferentially to the tumor with less than tenfold exposure in GI tissues, thus limiting gastrointestinal tract‐related toxicities.^[^ ^8a^ ^]^ JHU083 exhibited potent tumor inhibitory effects and increased survival in multiple experimental tumor models.^[^ ^6^, ^9^ ^]^ Remarkably, a heterogeneous immune response was evident upon glutamine antagonism. Both ex vivo and in vivo studies demonstrated that glutamine antagonism could skew CD8^+^ T cells toward an activated, long‐lived, memory‐like phenotype.^[^ ^6^ ^]^ Furthermore, JHU083 has the ability to transform immune‐suppressive myeloid‐derived suppressor cells (MDSCs) and tumor‐associated macrophages (TAMs) into tumor‐destructive proinflammatory macrophages.^[^ ^10^ ^]^ Interestingly, anti‐tumor effects were greatly diminished in immune‐deficient mice upon JHU083 treatment. Moreover, combining JHU083 with PD‐1 checkpoint blockade resulted in 90% response rates versus 0% with immunotherapy alone.^[^ ^6^ ^]^

Preventive vaccines are administered to individuals with or without premalignant lesions to activate adaptive immune responses before the onset of new or recurrent malignant lesions and to eliminate mutant cells prior to tumor formation. This is particularly important for individuals at high risk of developing lung cancer (e.g., smokers and former smokers), and those with resected primary lung cancer at high risk for relapse. Given JHU083's effects on antigen‐independent inhibition of glutamine addiction in tumor cells and its surprising enhancement of anticancer immunity, combining JHU083 with tumor antigen peptide vaccination is warranted. We previously developed an MHC class II‐restricted multi‐peptide vaccine (EVax) that targets both mouse and human wildtype EGFR.^[^ ^11^ ^]^ EVax is thus designed to enhance EGFR protein‐specific CD4^+^ T cell activation and recruit anti‐tumor effector immune cells to tumor sites. A striking decrease in EGFR‐driven lung tumorigenesis was observed in the EGFR^L858R^ transgenic mouse model when mice were treated with EVax before the induction of EGFR oncogene expression. However, since EGFR mutations exist in high‐risk patients long before malignancy formation, it is more clinically relevant to test the anti‐tumor effects of agents in mice after oncogene induction. The current study is the first to explore the efficacy of JHU083, an orally active small molecule prodrug of DON designed for preferentially activated in the TME through cleavage by esterases and peptidases, for its anti‐tumor effects in a spontaneous genetically engineered murine primary lung tumor model. The combined effects of JHU083 and EVax treatment on anti‐tumor immunity were investigated using flow cytometry and single‐cell RNA‐sequencing (scRNA‐seq) analyses of treated lung tumors.

## 2 Results

### 2.1 JHU083 Treatment Improves the Anti‐Tumor Efficacy of EVax

**Figure 1:**
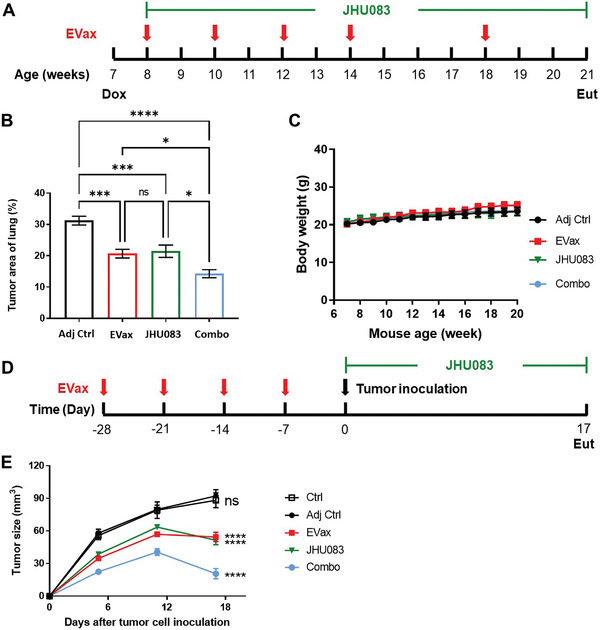
JHU083 enhanced the anti‐cancer efficacy of EVax in an EGFR^L858R^‐driven transgenic lung cancer model and a syngeneic EGFR^L858R^ lung tumor cell line model. A) Experimental design outlining the timing of Dox induction, JHU083 administration, vaccination, and experimental endpoint. B) Percent of lung tumor area at the experimental endpoint for each group (*n* = 9 per group). C) Average group body weights throughout the primary lung model study. D) Experimental design for the syngeneic lung tumor model. E) UN‐SCC680‐EGFR^L858R^ tumor development in all experimental groups (*n* = 6 per group). Data are shown as the mean ± SE; *p < 0.05, **p < 0.01, ***p < 0.001, and ****p < 0.0001; one‐way ANOVA for group comparisons.

We tested whether JHU083 could potentiate EVax's therapeutic effect in inhibiting lung cancer development in an EGFR^L858R^ mutation‐driven primary lung cancer model. EVax was started 1 week following EGFR^L858R^ transgene induction, followed by periodic boost vaccinations at indicated time points shown in **Figure** **1**A. JHU083 treatment was initiated at the same time via oral gavage and continued until the end of the study (Figure 1A).^[^ ^6^ ^]^ Anti‐tumor effects were determined by analysis of tumor load, which was assessed by measuring the percent tumor area in the Hematoxylin and Eosin (H&E) staining slides processed from lungs collected 14 weeks post doxycycline induction across all four groups. Compared to the adjuvant‐only control group, EVax and JHU083 treatment alone resulted in a decrease in tumor load by ≈33% and ≈31%, respectively. Tumor load was further decreased by EVax/JHU083 combined (Combo) treatment, resulting in ≈54% reduction relative to the adjuvant control, and significantly lower tumor load than either treatment alone (Figure 1B). No body weight changes were observed indicating lack of toxicity from the treatments (Figure 1C).

Next, we verified the additive anti‐tumor effects of this combination in an EGFR mutant lung tumor syngeneic model. In this model, wildtype A/J mice were given a total of four weekly EVax treatments (Figure 1D). 1 week after completion of the four vaccines, UN‐SSC680 tumor cells^[^ ^12^ ^]^ over‐expressing mutant EGFR^L858R^ protein (UN‐SCC680^L858R^) were subcutaneously inoculated into the abdomens of vaccinated mice, and JHU083 oral gavage treatment was initiated. Upon termination of the study, we found that treatment with EVax or JHU083 alone significantly decreased tumor sizes by 38% and 42%, respectively (Figure 1E). The combined treatment further increased the tumor growth inhibition to 77% (Figure 1E), and tumors were significantly smaller than those in mice given either treatment alone.

### 2.2 JHU083 and EVax Stimulate the Infiltration of Anti‐Tumor CD8^+^ Effector T Cells into Tumor‐Bearing Lungs

**Figure 2:**
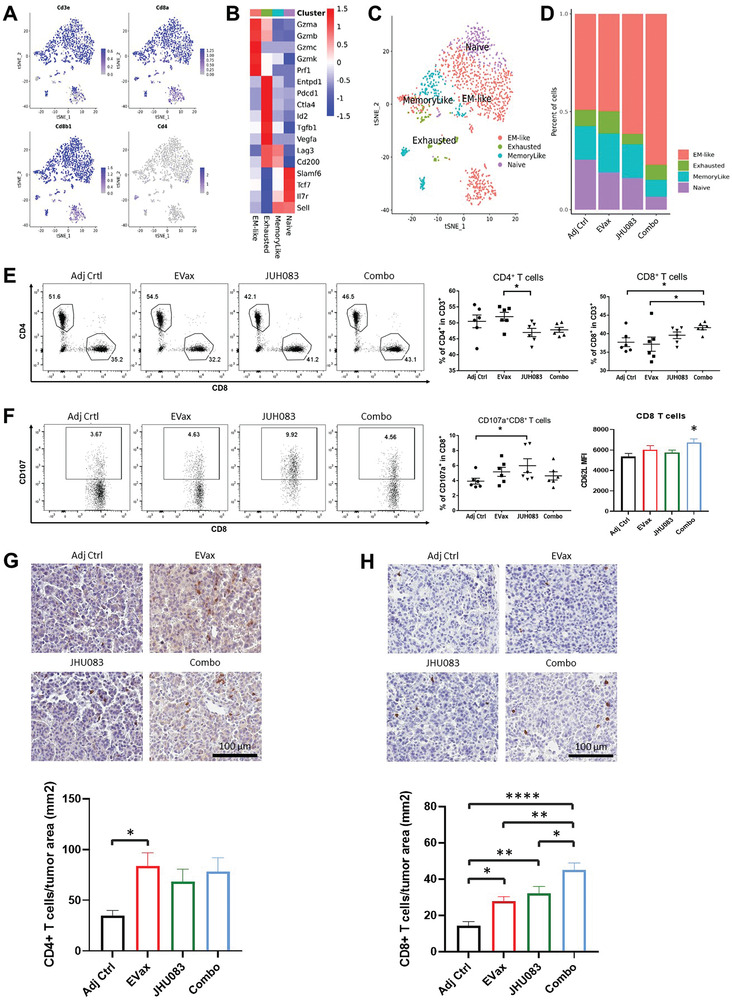
JHU083 treatment stimulated anti‐tumor CD8^+^ effector T cells within tumor‐bearing lungs. A) Canonical markers’ expression singled out CD8^+^ T cells based on scRNA‐seq data. B) Heatmap of the markers for each CD8^+^ T cell subset. C) The distribution of each CD8^+^ T cell subset. D) Percent changes in CD8^+^ T cells subsets across different treatment groups. E) Representative flow cytometry histograms and overall percentages of CD4^+^ (lower left panel) and CD8^+^ T cells (lower right panel) in the TME (*n* = 6 per group). F) Representative flow cytometry histograms and percentages of CD107a^+^CD8^+^ subpopulation within CD8^+^ T cell compartment under EVax peptides stimulation; CD107a^+^CD8^+^ cell counts under PMA/ionomycin stimulation (*n* = 6 per group). G) Representative IHC staining and quantitative data for tumor‐infiltrating CD4^+^ T cells from the EGFR^L858R^ model lung tumor. H) Representative IHC staining and quantitative data for tumor‐infiltrating CD8^+^ T cells from the EGFR^L858R^ model lung tumors. Data are shown as the mean ± SE; *p < 0.05, **p < 0.01, ***p < 0.001, and ****p < 0.0001; one‐way ANOVA for group comparisons.

Next, we tested whether the tumor inhibitory effects facilitated by glutamine antagonism involved activation of tumor‐specific immune responses by scRNA‐seq and flow cytometry. Studies have shown a positive correlation between elevated infiltration of cytotoxic CD8^+^ T cells into tumors and improved prognosis.^[^ ^13^ ^]^ Unsupervised clustering of CD8^+^ T cells identified in total CD45^+^ cells isolated from the lungs of tumor‐bearing mice revealed the presence of four clusters of CD8^+^ T cell subsets with distinct transcriptomic profiles (**Figure** **2**A–C). A heatmap of marker genes for the four CD8^+^ T subsets is shown in Figure 2B: a) Effector‐memory (EM‐like) CD8^+^ T cells, characterized by expression of cytotoxicity genes including Gzmb, Gzmk, and Prf1, and a lack of inhibitory receptors (e.g., Pdcd1 and Ctla4); b) Exhausted CD8^+^ T cells, which were characterized by expression of inhibitory receptors such as Pdcd1 (PD‐1), Entpd1 (Cd39), and Ctla4, and high expression of cytotoxic molecules (e.g., Gzma, Gzmb, and Prf1); c) Memory‐like CD8^+^ T cells, characterized by expression of inhibitory receptors (e.g., Lag3, CD200, but not Entpd1) and memory‐related genes (e.g., Tcf7, Il7r, and Sell); d) Naive CD8+ T cells, characterized by high expression of memory‐related genes (e.g., Tcf7, Il7r, and Sell), but not cytotoxicity genes or inhibitory receptors. The percentages of effector CD8^+^ T cells (EM‐like) were increased by both JHU083 treatment and to a larger extent by the JHU083/EVax combined (combo) treatment (Figure 2D). Results of the flow cytometry studies showed that the combo treatment significantly increased CD8^+^ T cell frequencies with no change in CD4^+^ T cell frequencies, as compared to the adjuvant control group (Figure 2E). After EGFR‐peptides stimulation in vitro, we observed that JHU083 treatment also significantly increased the frequency of CD107a^+^ CD8^+^ T cells, which represents activated degranulating cytotoxic CD8^+^ T cells (Figure 2F). However, we did not notice a further increase in CD107a^+^ CD8^+^ T cell percentages in the combined treatment group. To dissect the possible reasons behind this, we analyzed the mean fluorescence intensity (MFI) of the T cell memory marker (CD62L) among all treatment groups. We found a significant increase in CD62L MFI in the combination group compared with the Adj Ctrl group. Therefore, one possible reason behind this lack of increase in CD107a^+^ CD8^+^ T cell percentages in the combined treatment group could be due to the presence of more memory‐like CD8 T cells observed at the end of this long‐term study (Figure 2F). We further performed immunohistochemistry (IHC) staining for CD4^+^ T cells and CD8^+^ T cells in lung tumor tissues. We found that EVax treatment increased both CD4^+^ T and CD8^+^ T cell infiltration, while JHU038 increased CD8^+^ T cell numbers in tumor tissues. The biggest enhancement of CD8^+^ T cell infiltration was seen in the combination treatment group (Figure 2G,H).

### 2.3 JHU083 Improves EVax's T Cell Immunity by Increasing Anti‐Tumor Effector Th1 Cell Responses and Decreasing Pro‐Tumor Th17 Cells and Tregs in Tumor‐Bearing Lungs

**Figure 3:**
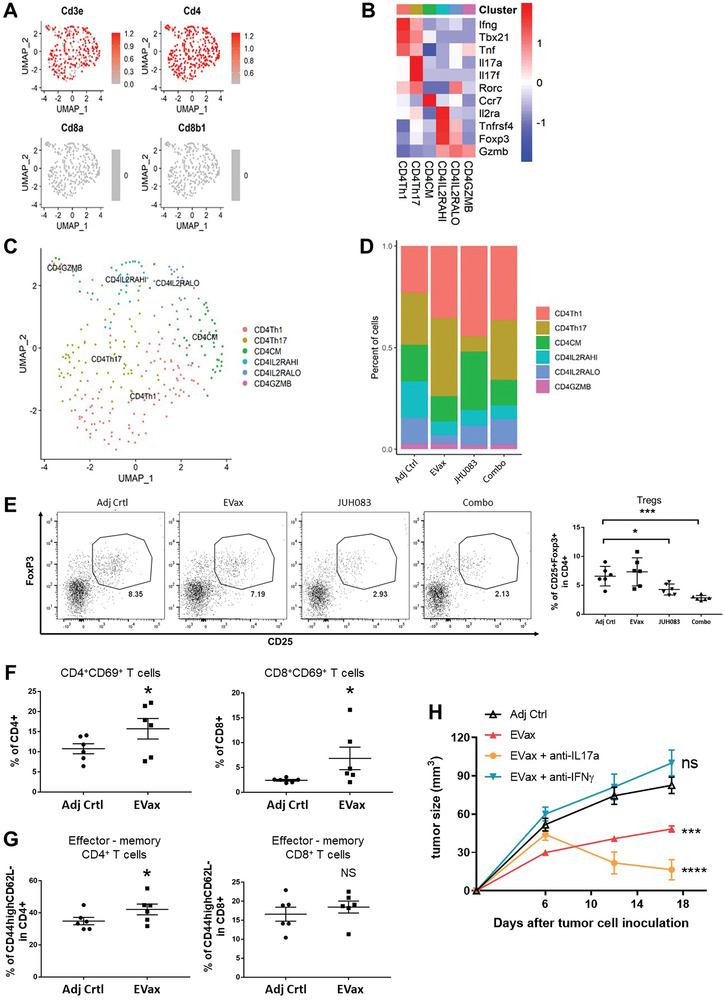
Changes in CD4^+^ T cell subsets across treatment groups. A) Canonical marker expression identified CD4^+^ T cells using scRNA‐seq data. B) Heatmap of markers for each subdivided CD4^+^ T cell subpopulation. C) The distribution of each CD4^+^ T cell subset. D) Percentage changes of CD4^+^ T cells subpopulations across different treatment groups. E) Foxp3^+^CD25^+^ Treg percentages across all groups by flow cytometric analysis (*n* = 6 per group). F,G) Combined flow cytometry results for gated CD4^+^ or CD8^+^ T cells stained with CD69, CD44, and CD62L to assess activated and effector‐memory T cells. H) UN‐SCC680‐EGFR^L858R^tumor development in all experimental groups upon in vivo neutralization of IL17a or IFN*γ* (*n* = 6 per group). Data are shown as the mean ± SE; *p < 0.05, **p < 0.01, ***p < 0.001, and ****p < 0.0001; two‐tailed student *t*‐test for two group comparison, and one‐way ANOVA for group comparisons.

In order to better understand the heterogeneity of the CD4^+^ T cell subsets present in tumor‐bearing lungs, scRNA‐seq was performed. First, we identified the CD4^+^ T cells using canonical CD4 markers, while excluding the CD8^+^ T cells (**Figure** **3**A). Next, we identified six CD4^+^ T cell subsets within the mouse lung tumors, similar to those identified before as being involved in anti‐tumor efficacy (Figure 3B,C).^[^ ^14^ ^]^ CD4^+^ Th1 cells overexpressed the marker genes Ifng, Tbx21, and Tnf;^[^ ^15^ ^]^ CD4 Th17 T cells overexpressed Il17a, Il17f, and Rorc marker genes;^[^ ^15^ ^]^ the two regulatory T cell (Treg) states, CD4IL2RAHI and CD4IL2RALO, showed high and low expression of Il2ra, respectively, and both showed high expression of Foxp3. CD4GZMB T cells showed high expression of Gzmb but did not express Foxp3.^[^ ^10^ ^]^ CD4^+^ central‐memory T cells (CD4CM) were identified by expression of Ccr7.^[^ ^14^ ^]^ We observed significant changes in percentages of CD4^+^ T cell subsets across the different treatment groups (Figure 3D). Effector CD4^+^ Th1 cell subset percentages were increased in both the EVax and JHU083 treatment groups. The Th17 subset was increased in the EVax group but decreased in the JHU083 treatment group. CD4CM cell abundance was increased by the JHU083 treatment. Total Treg cell abundance (IL2RAHI+IL2RALO) was decreased in all three treatment groups. Reductions of total Treg frequencies in both JHU083‐ and combo‐treated mice were also observed by flow cytometry, with the combo treatment inducing the most significant change (Figure 3E). Separately, CD4IL2RAHI Treg abundance was decreased in each of the three treatment groups; the CD4IL2RALO Treg subset decreased most in EVax‐treated mice.

In the flow cytometry studies, we found that frequencies of CD69^+^ and CD44^hi^ CD62L^low^ effector‐memory CD4^+^ T cells were enhanced in the lung tumors of mice given EVax treatment, as compared to the adjuvant group (Figure 3F,G). These results suggested that activated CD4^+^ effector T cells are involved in the EVax‐mediated anti‐tumor response. Activated CD4^+^ Th1 T cells have been shown to exert anti‐tumor effects against various cancer models via cytokines, such as interleukin‐2 (IL‐2), tumor necrosis factor‐*α* (TNF‐*α*), and interferon‐*γ* (IFN‐*γ*).^[^ ^16^ ^]^ However, the role of Th17 cells in cancer immunity is unclear.^[^ ^17^ ^]^ In a primary tumor model, we found that both Th1 and Th17 cell populations were expanded after EVax treatment (Figure 3D). To better understand the role of these CD4^+^ T cell subsets in tumor protection induced by EVax immunization, we examined the impact of neutralizing IFN‐*γ* (the major cytokine player of Th1 cells) and IL‐17a (the major cytokine player of Th17 cells) in EVax‐treated mice (Figure 3H). Although EVax strongly inhibited tumor growth, as expected, the EVax anti‐tumor response was eliminated in mice treated with anti‐IFN‐*γ* (Figure 3H), indicating that IFN‐*γ* plays a critical role in the EVax‐mediated anti‐tumor response. Surprisingly, EVax‐mediated tumor growth inhibition was further increased in mice treated with anti‐IL‐17a (Figure 3H). Together, these results indicate that Th1 cells, but not IL‐17a‐producing cells (including Th17 cells), are involved in the anti‐tumor response generated by EVax treatment in our tumor model.

**Figure 4:**
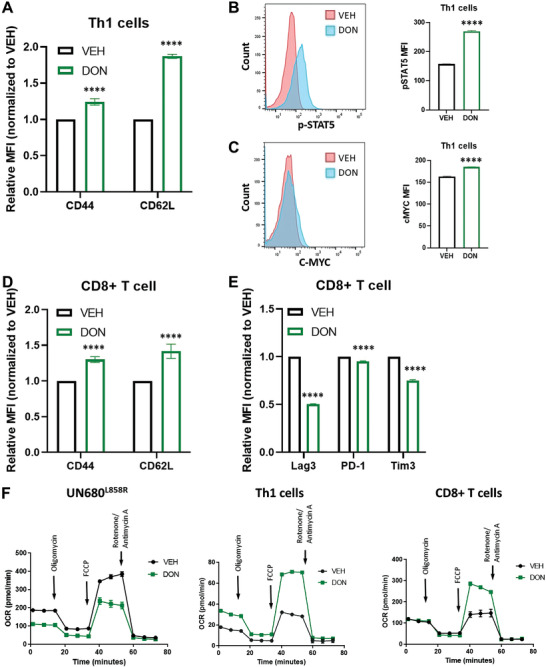
Functional and metabolic changes in Th1 and CD8 T cells in presence of glutamine antagonism. A–C) Naïve CD4^+^ T cells were activated and differentiated in the presence of vehicle or DON (1 µm) for 3 days and rested in IL‐2 for two additional days with vehicle or DON, and analyzed by FACS for activation and memory markers (A) and p‐STAT5 (B) and c‐MYC (C). D,E) Naïve CD8^+^ T cells were activated in the presence of vehicle or DON (1 µm) for 2 days and rested in IL‐2 for two additional days with vehicle or DON, and analyzed by FACS for activation and memory markers (D) and exhaustion markers (E). F) Metabolic flux analyses of tumors cells, Th1 cells, and CD8 T cells in the presence of vehicle or DON (1 µm) in vitro. Data are shown as the mean ± SE; *p < 0.05, **p < 0.01, ***p < 0.001, and ****p < 0.0001; two‐tailed student *t*‐test for two group comparisons.

Encouraged by our in vivo findings that glutamine inhibition could expand Th1 cells in addition to the previously published CD8^+^ T cell expansion in tumor‐containing lungs, we assessed the cell‐intrinsic nature of this finding on Th1 cells. We activated and expanded naïve CD4^+^ T cells with anti‐CD3, anti‐CD28, IL‐2, IL‐12, and IL‐14 in the presence or absence of DON. Flow cytometry analysis revealed that Th1 cells cultured in the presence of DON exhibit increased expression of the activation marker CD44 and memory marker CD62L (**Figure** **4**A). Consistent with previous findings that glutaminase (GLS) deprivation favors upregulation of the IL2‐STAT5/mTORC1/Myc signaling pathways, which are associated with Th1 cell differentiation,^[^ ^18^ ^]^ we also noted increased expression of p‐STAT5 and c‐MYC after DON treatment (Figure 4B,C). Moreover, we also found that DON‐treated CD8 T cells showed upregulation of CD44 and CD62L and downregulation of exhaustion markers Lag3, PD‐1, and Tim3 (Figure 4D,E), which is consistent with previous results.^[^ ^6^ ^]^ Considering the differential effects of glutamine antagonism to inhibit tumor cells while enhancing T cell function, we investigated the metabolic status of tumor cells, Th1 cells and CD8 T cells. Although DON exposure is known to greatly suppress oxidative phosphorylation (OXPHOS) in cancer cells, DON robustly enhanced OXPHOS during Th1 activation and differentiation (Figure 4F). In addition, we also observed increased OXPHOS in DON‐treated CD8 T cells (Figure 4F), which is in line with previous findings.^[^ ^6^ ^]^

### 2.4 Impact of Different Treatments on the Composition of Other Immune Cells

**Figure 5:**
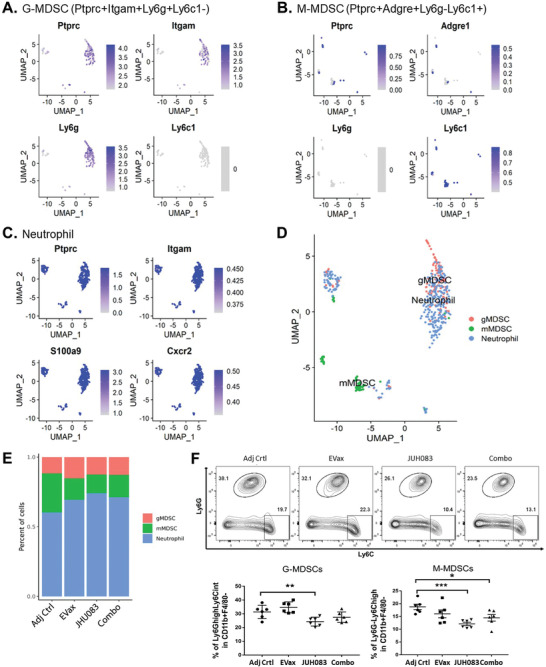
Changes in the G‐MDSC, M‐MDSC, and neutrophil cell populations across treatment groups in the EGFR^L858R^ transgenic mouse model. Expression of specific marker genes was used to identify: A) G‐MDSCs (Ptprc+Itgam+Ly6g+Ly6c1−), B) M‐MDSCs (Ptprc+Adgre+Ly6g−Ly6c1+), and C) neutrophils (Ptprc+Itgam+S100a9+Cxcr2+). D) The relative distribution of G‐MDSCs, M‐MDSCs, and neutrophils in tumor tissue. E) Proportion changes of G‐MDSCs, M‐MDSCs, and neutrophils across different treatment groups. F) MDSC percentage changes across all groups by flow cytometric analysis; representative histograms and combined data graphs are shown (*n* = 6 per group). Data are depicted as mean ± SE; *p < 0.05, **p < 0.01, ***p < 0.001, and ****p < 0.0001; one‐way ANOVA for group comparisons.

We next identified the three immune cell populations—granulocytic MDSCs (G‐MDSCs), monocytic MDSCs (M‐MDSCs), and neutrophils based on the expression of key marker genes (**Figure** **5**A–D).^[^ ^19^ ^]^ Further analyses showed that treatment with JHU083, EVax, or the combo decreased the percentages of M‐MDSCs (Figure 5E). Consistently, results of the flow cytometry analyses demonstrated a significant reduction in the percentages of both MDSC subsets by JHU083 treatment alone, with M‐MDSCs showing a greater reduction, from ≈18% to ≈11% (Figure 5F). The reduction of immunosuppressive M‐MDSCs in all three treatment groups suggests that inhibiting M‐MDSC accumulation in the tumor tissue may be another route by which EVax or JHU083 treatments contribute to the anti‐tumor function.

**Figure 6:**
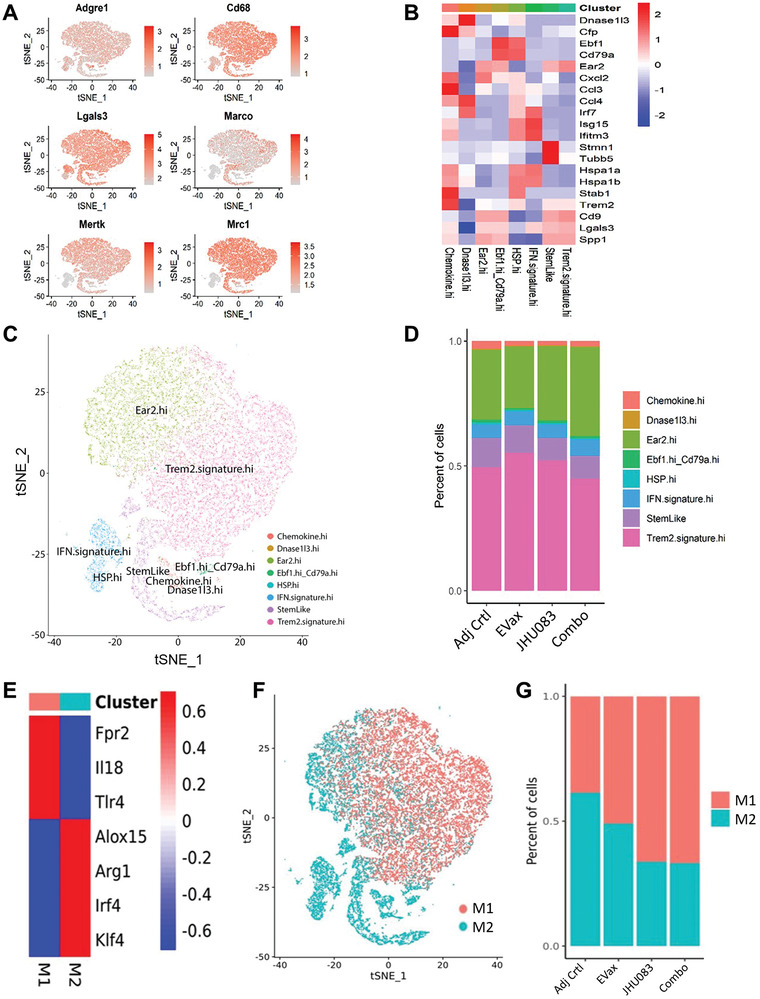
Effects of different treatments on macrophages subpopulations. A) Canonical markers were used to identify the macrophages based on scRNA‐seq data. B) Heatmap of the markers for each of the eight macrophages subpopulations. C) Plot of distribution of the eight macrophages subpopulations. D) Proportion changes in the macrophages subpopulations across different treatment groups. E) The marker gene expression was used to identify the M1 and M2 populations. F) The overall clustering of the macrophages into M1 and M2 subpopulations. G) The percentages of M1/M2 macrophage populations in the adjuvant and different treatment groups.

We also performed deep clustering of macrophages to see if there were changes in macrophage subpopulations after JHU083 or EVax treatment. First, macrophages were identified using canonical marker genes including F4/80 (Adgre1), Marco, Mertk, and Mrc1 (**Figure** **6**A). Second, we performed deep clustering of macrophages and identified eight previously identified macrophage subsets,^[^ ^20^ ^]^ including Chemokine.hi, Dnase1l3.hi, Ear2.hi, Ebf1.hi_Cd79a.hi, HSP.hi, IFN.signature.hi, StemLike, and Trem2.signature.hi (Figure 6B,C). These subsets were similar to either anti‐tumor M1 or pro‐tumor M2 macrophages. Although no major changes in the treatment groups were found, we made a number of observations (Figure 6D). Ear2.hi M*φ*, a type of activated M2‐like tumor‐promoting M*φ*, was decreased by EVax vaccination. StemLike M*φ*, transcriptionally similar to cancer stem cells, decreased in the JHU083 and/or EVax treatment groups. The percentage of pro‐tumorigenic Trem2.signature.hi M*φ* was reduced by the combo treatment. We also divided macrophages into two major types—tumor‐resistant M1‐like and tumor‐promoting M2‐like macrophages. The results showed that proportions of M1‐like macrophages increased in treatment groups compared to the control group, and this increase was higher after JHU083 and the combo treatment (Figure 6E–G). These findings suggested that macrophage composition is altered toward anti‐tumorigenic macrophages by treatment with JHU083 and/or EVax.

**Figure 7:**
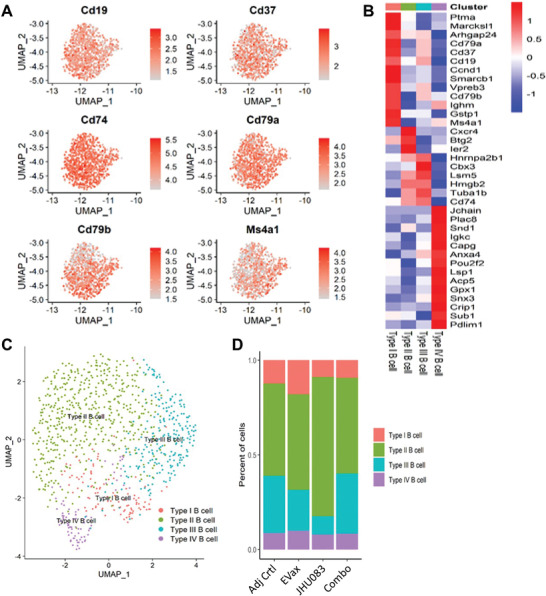
The effect of different treatments on the B cells subpopulations. A) Canonical markers were used to identify the B cells based on scRNA‐seq data. B) Heatmap of the markers for each of the four B cell subsets (Type I–IV). C) Distribution of each of the four B cell subsets. D) Proportion changes in the Type I–IV B cells subsets across different treatment groups.

We further investigated subpopulations of B cells present in tumor tissues of treated mice. According to a recent study, B cells can be divided into four subsets (Type I–IV).^[^ ^21^ ^]^ After separating B cells from other immune cells using unique B cell marker genes (**Figure** **7**A), we recapitulated the four types of B cells, as reported previously (Figure 7B,C).^[^ ^21^ ^]^ EVax treatment increased the proportion of Type I B cells (Figure 7D). JHU083 treatment increased the proportion of Type II B cells (Figure 7D). EVax or JHU083 treatment decreased the percentages of Type III B cells. Previous report showed that Type I and II B cells could affect immunogenicity and apoptosis associated with immune escape mechanisms of cancer cells (21). Type III B cells closely communicate with other cells and play a role in immunosuppression.^[^ ^21^ ^]^ The exact role of changes of Type I, II, or III B cells in mediating the anti‐tumor effects of EVax and JHU083 remains to be elucidated.

**Figure 8:**
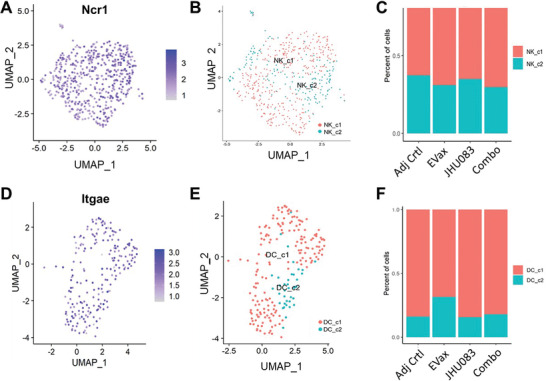
NK cell subset proportions and DC cell subset proportions across treatment groups. A) The canonical marker, Ncr1, was used to single out the NK cells based on scRNA‐seq data. B) NK cells were divided into two NK subsets (NK_c1 and NK_c2). C) Proportions NK_c1 and NK_c2 cell subsets across different treatment groups. D) Canonical marker, Itgae (CD103), was used to identify DCs based on scRNA‐seq data. E) DCs were divided into two subsets (DC_c1 and DC_c2). F) Proportions of DC subsets across different treatment groups.

Finally, natural killer (NK) cells were separated from other immune cells and two subclusters were identified (**Figure** **8**A,B). However, no significant changes in the two NK cells subsets across different treatment groups were observed (Figure 8C). Dendritic cells (DCs) were classified into two subpopulations, and no significant changes were observed in the frequencies of these two DC subsets after JHU083 or the combo treatment (Figure 8D–F).

### 2.5 JHU083 and Combo Treatment Altered Signaling Pathways in Immune Cells

**Figure 9:**
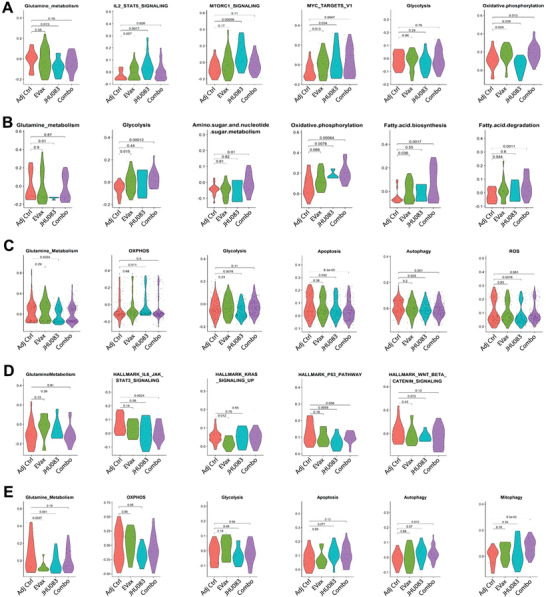
Significantly altered signaling pathways in distinct types of immune cells across the control and three treatment groups in the EGFR^L858R^ transgenic mouse model. Metabolic changes across the four groups were shown for A) CD4^+^ Th1 cells, B) CD4^+^ Th17 cells, C) EM‐like Cd8^+^ T cells, D) Tregs, and E) M‐MDSC cells.

Recent research showed that glutamine metabolism has distinct roles in promoting Th17 cells and inhibiting Th1 cells and that GLS inhibition can reverse such effects in inflammatory diseases.^[^ ^18^ ^]^ Since JHU083 is a glutamine metabolism inhibitor, our results demonstrated similar findings in an EGFR‐driven lung cancer model. Consistent with our in vitro results (Figure 4B), we observed upregulation of the IL2‐STAT5/mTORC1/Myc signaling pathways that are associated with Th1 cell differentiation in all three treatment groups (**Figure** **9**A). Coupled with downregulation of glutamine metabolism, mTORC1 signaling was elevated only in the JHU083 group, which may explain the largest increase in Th1 cells for this group, as enhanced mTORC1 activity has been directly linked to Th1 cell proliferation.^[^ ^18^ ^]^ As expected, glutamine pathway downregulation was observed in Th1 cells after JHU083 treatment. However, slight upregulation of the OXPHOS pathway was revealed in the JHU083 group, which is in line with our in vitro metabolic flux analyses of DON‐treated Th1 cells (Figure 4F). Interestingly, sc‐RNAseq also suggested that Th1 cells in combo‐treated mice have the highest level of OXPHOS activity. Glutamine metabolism has a pivotal role in Th17 cell generation and cytokine (IL‐17) secretion.^[^ ^18^, ^22^ ^]^ In Th17 cells (Figure 9B), glutamine metabolism was similarly downregulated in the JHU083 treatment group, which is in accordance with the robust reduction in Th17 lineage in JHU083‐treated mice. GLS inhibition may also limit the pro‐tumor IL‐17 secretion, which further potentiates the anti‐tumor effects of JHU083.

We further analyzed metabolic adaptations and death pathway changes in CD8^+^ T cells isolated from EGFR^mutant^ lung tumors after JHU083 or the combo treatment. Glutamine metabolism activity decreased in the JHU083 treatment group, which was accompanied by an increase in OXPHOS activity in the effector CD8^+^ T cells (e.g., “EM‐like”) and a decrease in glycolysis, apoptosis, autophagy, and ROS pathways (Figure 9C). In addition, the activity of cell death pathways including apoptosis, autophagy, and ROS was decreased by the combo treatment in EM‐like CD8^+^ T cells (Figure 9C). These observations help explain the increase of EM‐like CD8^+^ T cells in the JHU083 and combo treatment groups observed in Figure 2. Our results were in line with the previous findings that targeting glutamine metabolism in CD8^+^ T cells in the TME leads to metabolic reprogramming with enhanced survival, proliferation, and effector function.^[^ ^6^ ^]^

Tregs and M‐MDSCs were the two immunosuppressive cell populations whose abundance decreased in the JHU083 and/or EVax groups (Figures 3 and 5). In Treg cells, JHU083 treatment inhibited several oncogenic signaling pathways related to Treg immunosuppressive function such as Stat3, p53, and Wnt (Figure 9D). Similarly, the combo treatment inhibited Stat3 and p53 signaling, while EVax treatment inhibited Kras signaling in Treg cells (Figure 9D). In M‐MDSCs, JHU083 treatment inhibited glutamine metabolism and OXPHOS while it enhanced apoptosis and autophagy pathways (Figure 9E). Finally, the combo treatment promoted the cell death pathways autophagy and mitophagy (Figure 9E). These pathway activity alterations may account for the decrease in M‐MDSCs by the corresponding treatment.

To validate our sc‐RNAseq analysis results on Th1 cells, we performed differential gene expression and pathway analysis on RNA‐seq datasets from a public database—GSE112244. This study performed RNA‐sequencing (GSE112244) on Th1 cells treated with vehicle or CB839 (a GLS inhibitor). It was found that although GLS activity was inhibited, some signaling pathways were upregulated including OXPHOS, IL‐2/STAT5, MTORC1, and MYC (Figure S1A, Supporting Information). Pathways relating to glutamine transport were downregulated. These bulk RNA‐seq results available in a public database match our sc‐RNAseq findings in Figure 9A and our proteomic validation results shown in Figure 4B,C,F. To validate our sc‐RNAseq results on CD8^+^ T cells and M‐MDSCs, we performed differential gene expression and pathway analysis of RNA‐seq datasets from two previous studies.^[^ ^6^, ^14^ ^]^ The first study performed RNA‐sequencing (GSE120345) on sorted CD8^+^ TILs (tumor‐infiltrating lymphocytes) from vehicle or JHU083 treated MC38 tumor‐bearing mice.^[^ ^6^ ^]^ It was found that JHU083 treatment caused upregulation of OXPHOS and downregulation of glutamine metabolism, apoptosis, ROS, autophagy, and glycolysis in CD8^+^ TILs (Figure S1B, Supporting Information), which supported our observation of similar pathway activity changes in EM‐like CD8^+^ T cells (Figure 9C). The second study used RNA‐sequencing (GSE119733) data from sorted TAMs from 4T1 mouse breast tumor bearing mice with or without JHU083 treatment.^[^ ^10^ ^]^ The TAMs showed downregulation of glutamine metabolism, OXPHOS, and glycolysis pathways, and upregulation of apoptosis and autophagy pathways (Figure S1C, Supporting Information), which supported our findings in the M‐MDSC cells (Figure 9E). The downregulations of multiple pathways in TAM cells reported in the initial paper^[^ ^10^ ^]^ were also recapitulated in our analysis.

## 3 Discussion

One significant metabolic feature of tumor cells is their reliance on an exogenous supply of glutamine. Glutamine is versatile enough to provide the anaplerotic nitrogen and carbon sources for the biosynthesis of many macromolecules (nonessential amino acids and nucleotides) and replenishment of metabolic intermediates in the Kreb's cycle.^[^ ^2^ ^]^ Glutamine metabolites also participate in the biosynthesis of major anti‐oxidants to protect cancer cells from experiencing oxidative stress.^[^ ^23^ ^]^ Hence, most tumor cells cannot survive in the absence of glutamine. Many attempts have been made to target glutamine metabolism for cancer treatment, and promising anti‐tumor activities have been observed with its specific inhibitors in preclinical models.^[^ ^24^ ^]^ As with tumor cells, however, activated T lymphocytes rely heavily on glutamine uptake and metabolism, and depleting glutamine from T cells can significantly hamper T cell proliferation and effector functions.^[^ ^25^ ^]^ Therefore, one can envision that rapidly proliferating tumor cells can outcompete immune cells for limited glutamine in the TME, resulting in suppressed T cell anti‐tumor immunity. EGFR activation, induced by mutations, has been observed to induce upregulation of glutamine metabolism in some cancer studies.^[^ ^4^, ^26^ ^]^ A strategy to inhibit GLS 1 in addition to EGFR block has shown promising anti‐tumor effects in preclinical colon and lung cancer models.^[^ ^4^, ^27^ ^]^ Therefore, we hypothesized that tumors with glutamine addiction, might benefit from preferential glutamine inhibition in the TME, and thereby, enhance the efficacy of EVax against EGFR‐driven lung tumorigenesis.

Unlike systemic glutamine antimetabolites that caused unacceptable toxicity in clinical trials, a more selective approach using the prodrug of L‐DON, JHU083, is less toxic and thus more ideal for treating cancer patients or using as an immunomodulatory agent for cancer prevention in high‐risk but relatively healthy individuals. Our study is the first to test JHU083 in an oncogene‐induced murine primary lung tumor model that has similar molecular traits to human lung cancer. In this model, long‐term oral administration of JHU083 induced no apparent toxicities such as body weight loss or other clinical signs. In line with previous preclinical studies,^[^ ^6^, ^9^ ^]^ we found that JHU083 alone mediated beneficial anti‐tumor effects in a mutant EGFR primary lung tumor model, demonstrating ≈30% inhibition of tumor growth. In addition, this is the first study that examines the potential effects of a cancer vaccine plus glutamine antagonism combination in a lung tumor model. After combining JHU083 with EVax, a peptide vaccine that specifically targets wildtype regions of the EGFR protein, the inhibitory rate increased to around 54%, indicating JHU083 enhanced the anti‐tumor efficacy of EVax. The studies employing the EGFR mutant tumor syngrafts similarly showed that both the JHU083 and the EVax were effective in reducing tumor growth. The combined effect implies that interactions between the immune cells elicited by the vaccine and the altered TME were caused by JHU083. Our data advocates for testing this novel combination of anti‐tumor agents in the clinic as a new strategy for inhibiting EGFR‐driven lung cancer progression.

The TME refers to the cellular/molecular environment surrounding tumor cells. TME is comprised of immune cell populations (lymphocytes, B cells, NK cells, macrophages, DCs, MDSCs, etc.), blood vessels, extracellular matrix, and fibroblasts.^[^ ^28^ ^]^ Ample evidence supports the critical role of cytotoxic EM‐like CD8^+^ T cells in tumor immunosurveillance.^[^ ^29^ ^]^ The TME within EGFR‐mutation driven lung tumors has been depicted as having a lack of anti‐tumor lymphocytes and an accumulation of immune‐suppressive factors (cells and cytokines).^[^ ^30^ ^]^ One recent study revealed that there is a positive correlation between EGFR overexpression and significant lack of cytotoxic CD8^+^ T cells in TME.^[^ ^31^ ^]^ Moreover, it was revealed that EGFR mutations could actively promote CD8^+^ T cell apoptosis more than wildtype non‐small cell lung cancer patients.^[^ ^32^ ^]^ A recent study showed that JHU083 could shut down bioenergetic pathways in cancer cells while boosting effector CD8^+^ T cell anti‐tumor responses to overcome immune evasion.^[^ ^6^ ^]^ We hypothesized that JHU083 might synergize with the EVax to relieve immune suppression and lead to enhanced anti‐tumor immune responses. Interestingly, JHU083 drives the expansion of total CD8^+^ T cells as well as the cytotoxic effector‐memory like CD8^+^ T cells in lung tumors. Notably, the anti‐cancer efficacy of JHU083 treatment was completely lost in the absence of C CD8^+^ T cells, indicating a crucial role for CD8^+^T cell‐mediated immunity. Although glutamine pathway genes were downregulated, we observed an increase in OXPHOS activity and a decrease in the apoptosis, autophagy, and ROS pathways in EM‐like CD8^+^ T cells after JHU083 treatment. These changes were consistent with previous findings when JHU083 was tested in vitro.^[^ ^6^ ^]^ By combining with EVAX, we noticed a further increase in CD8^+^T cell infiltration in EGFR mutation‐driven lung tumors, which may be the cause for improved efficacy.

Th1 cells are regarded as the most important helper T cell type in anticancer immunity, whereas Th17 cells are deemed as having dual roles in tumor immunity.^[^ ^33^ ^]^ IL17, the main cytokine produced by Th17 cells, induces inflammatory mediators and may engage myeloid cells to promote cancer progression.^[^ ^33^ ^]^ One recent study suggested that glutamine metabolism limited Th1 effector cell differentiation while polarizing Th17 effectors cell differentiation.^[^ ^18^ ^]^ GLS inhibition promotes Th1 expansion by sensitizing Th1 cells to IL‐2‐mediated mTORC1 signaling while constraining Th17 cell proliferation.^[^ ^18^ ^]^ Interestingly, we also found that treatment with JHU083, which also inhibits GLS, increased frequencies of the anti‐tumor Th1 cells while inhibiting the pro‐tumor Th17 cells. ScRNA‐seq studies also identified the upregulation of *Il‐2, Wnt, Mtor1*, and *Myc* genes, which is consistent with previous findings when GLS is inhibited.^[^ ^18^ ^]^ Interestingly, glutamine pathway inhibition not only boosts CD8^+^ T cell OXPHOS but also triggers OXPHOS in Th1 cells, which is in accordance with the metabolic characteristics of a T cell memory phenotype. A higher level of OXPHOS allows memory T cells to quickly activate upon encountering an antigen. Induction of memory T cells by JHU083 may facilitate the generation of long‐term immunity after vaccination. We also found that our MHC class II‐restricted peptide vaccine, EVax, not only has an anti‐tumor Th1 cell‐promoting effect but also induced pro‐tumor Th17 cells. Prior reports have suggested that IL‐17 inhibition helps augment tumor immunity by enhancing cytotoxic CD8^+^ T cells and Th1 cells in the TME.^[^ ^34^ ^]^ Therefore, after combining GLS inhibition with EVax vaccination, we noticed a CD4^+^ T cell landscape with anti‐tumor Th1 cells as the major cell type.

Increases in effector CD8^+^ T cells and Th1 effector cells after JHU083 treatment coincided with significant decreases in the frequencies of major immunosuppressive populations of cells including Tregs and MDSCs. A recent study indicated that treatment with the glutamine transport inhibitor V‐9302 could reduce percentages of CD4^+^FoxP3^+^CD25^+^CD127^low^ Tregs in human tumor tissues.^[^ ^35^ ^]^ Glutamine metabolism has also been reported to be critical for the recruitment and functional activity of MDSCs.^[^ ^36^ ^]^ Glutamine antagonism has successfully resulted in reduced MDSC infiltration into the TME.^[^ ^10^, ^36^ ^]^ We found that JHU083 inhibited M‐MDSCs in our tumor model. The inhibition of glutamine metabolism and OXPHOS enhanced apoptosis and autophagy pathways, which could be the potential reason for the decrease in M‐MDSCs.

Recently, a great deal of attention has been directed to targeting glutamine metabolism for cancer treatment. Broad glutamine metabolism inhibition with DON prodrugs such as JHU083 and Sirpiglenastat (DRP‐104) (Dracen Pharmaceutical's clinical agent), may hold a lot of promise for the treatment of solid tumors. It is noteworthy that there is a clinical stage glutamine antagonist prodrug termed DRP‐104 that is being developed by Dracen Pharmaceuticals, which is in clinical studies (ClinicalTrials.gov Identifier: NCT04471415). Our data suggest that glutamine antagonism is a rational and effective strategy for combination therapy with other immune‐augmenting modalities (e.g., vaccination) to generate improved anti‐tumor efficacy. We found that JHU083 improves immune surveillance by decreasing immunosuppressive components of the immune response (MDSCs, Tregs, and Th17 cells) while increasing effector CD8^+^ T cells and Th1 cells. Importantly, JHU083 does not appear to promote the development of immune‐associated or GI‐related toxicities. No small molecule compound targeting immunometabolism has yet been approved for cancer treatment. Our data support advancing the use of JHU083 as adjuvant therapy for EGFR‐mutant NSCLCs, and more importantly, our data indicate the potential usage of a JHU083/EGFR‐Vax combinatorial approach for primary (L858R‐mediated tumorigenesis) prevention in high‐risk patients.

In summary, the present study demonstrates for the first time that glutamine blockade with JHU083 significantly enhances the efficacy of EVax in controlling the development and growth of EGFR‐driven lung cancer. This is highly significant because although we have shown that EVax is effective in preventing EGFR‐driven lung tumorigenesis in a pure prevention setting, EVax is much less effective in mice carrying existing premalignant lesions. Thus, combining EVax with JHU083, an orally bioavailable glutamine antagonist prodrug will provide optimal efficacy to prevent EGFR‐driven lung cancer development and progression. Second, the TME in EGFR mutation‐driven lung cancer is poorly immunogenic, lacks infiltration of anti‐cancer immune effector cells, and shows poor response to anti‐PD‐1/PD‐L1 treatment. We demonstrate that glutamine antagonism not only increases anti‐tumor CD8^+^ T cells infiltration of EGFR‐mutated lung tumors, but JHU083 can also significantly potentiate Th1 cell expansion, activation, and memory formation which have not been shown previously. GLS deficiency changes the metabolic features of Th1 toward an oxidative phenotype. Upregulation of IL‐2‐mediated mTORC1 signaling appears to play a role in Th1 differentiation during glutamine inhibition. Glutamine blockade with JHU083 induces robust anti‐tumor immune responses in “uninflamed” and poorly immunogenic EGFR‐driven lung cancer, and JHU083 may enable EGFR‐driven lung cancer more amenable to immunoprevention and immunotherapies. Finally, this study found that JHU083 could significantly decrease the presence of immunosuppressive cells including MDSCs, Tregs, and pro‐tumor Th17 cells. In combination with EVAX, JHU083 further decreases Tregs and increases the infiltration of anti‐tumor CD8^+^ T cells into EGFR‐mutated lung tumors. These new observations significantly expand our previous knowledge of JHU083 various cell types in the TME.

## 4 Experimental Section

### EVax Preparation and Immunization

Two peptides representing human EGFR residues 306–325 (SCVRACGADSYEMEEDGVRK) and residues 897–915 (VWSYGVTVWELMTFGSKPY) were identified using previously published methods.^[^ ^37^ ^]^ The peptides were synthesized by Genemed Synthesis Inc. (San Antonio, TX), and their purity (>95%) was verified by HPLC. The vaccine was prepared according to previously published methods.^[^ ^11^ ^]^ Mice were vaccinated with 50 µg of each peptide. The adjuvant used in this study was complete Freund's adjuvant (CFA; only the first dose) or incomplete Freund's adjuvant (IFA; the following doses). Adjuvant or adjuvant/peptides mixtures were subcutaneously injected in a bolus of 100 µL bilaterally behind the shoulders and in front of the hind legs, in the schedule indicated in Figure 1A.

### Human EGFR Mutations Were Overexpressed in UN‐SCC680 Cells

For the syngeneic tumor model, a mouse lung cancer cell line (UN‐SCC680) was developed to overexpress human EGFR mutations. pBabe EGFR(L858R) plasmids were obtained from Addgene (Cat: # 11012). pBabe EGFR(L858R) lentivirus particles were obtained by co‐transfecting pBabe EGFR(L858R) with packaging plasmids (pMD2 and pAX2) into HEK293T cells. UN‐SCC680 mouse lung squamous cell carcinoma cells were infected with concentrated pBabe EGFR(L858R) lentivirus particles and then were screened by puromycin to obtain a stable EGFR(L858R)‐overexpressed UN‐SCC680 cell line. The protein expression of L858R was detected by western blot analysis. Cells were then expanded by culturing in RPMI‐based media supplemented with 1 µg mL^−1^ puromycin for the selection and maintenance of cells with transgene for following in vivo studies.

### Mouse Models and Treatments

Transgenic mice expressing mutant L858R human EGFR on a C57bl/6 (B6) background were a generous gift from Dr. William Pao (Vanderbilt University) and Dr. Katerina Politi (Yale University). To facilitate lung‐specific inducible expression of the transgene, EGFR^L858R^ mice (C57bl/6 strain) were crossed with mice expressing the Tet‐on Clara Cell Secreted Protein (CCSP) on the C57bl/6 background, and only the F1 generation that harbored both EGFR L858R and CCSP was used. All mice were housed in the Biomedical Resource Center at the Medical College of Wisconsin (MCW), Milwaukee, WI. All procedures were approved by the MCW IACUC.

For primary lung cancer experiments, 7 week‐old female B6 mice were divided into the following four groups: 1) theadjuvant control mice that received CFA/IFA; 2) EVax‐treated mice that received the two EGFR peptides suspended in adjuvant; 3) mice treated with JHU083 (0.2 mg kg^−1^ in PBS) by oral gavage 5 times per week;^[^ ^6^ ^]^ and 4) combined treatment with EVax/adjuvant plus JHU083. Doxycycline‐containing diets (TD. 01306, Envigo Teklad) were administrated as food pellets starting at week 7. Vaccination was given 1 week post doxycycline induction, followed by four more vaccinations at 2‐week intervals and an additional boost vaccine 4 weeks later. JHU083 gavage treatments were started 1 week after the doxycycline induction of lung adenocarcinoma (Figure 1A).

For evaluation of anti‐tumor efficacy in the UN‐SSC680^L858R^‐derived syngeneic lung cancer model, wild‐type female A/J mice were purchased from the Jackson Laboratory. 5–6 week‐old mice were grouped according to similar bodyweights into the following five groups: 1) vehicle control mice that received 0.07% DMSO in PBS solution; 2) adjuvant control mice that received CFA/IFA once per week for a total of four doses; 3) Evax‐treated mice that received the two EGFR peptides suspended in adjuvant once per week for a total of four doses; 4) mice treated with JHU083 (0.2 mg kg^−1^ in PBS) by oral gavage in a 100 µL bolus four to five times per week; and 5) combined treatment, in which mice received both EVax and JHU083 (Figure 1A). UN‐SSC680^L858R^ cells were trypsinized, washed with PBS, and filtered through a 30‐µm strainer. Then, 5 × 10^6^ cells (in 0.1 mL of PBS) were inoculated subcutaneously into the abdomens of experimental and control mice 1 week after four vaccinations (Day 0). JHU083 was administered on days 1, 2, 3, 4, 5, 8, 9, 10, 11, 14, 15, and 16 after tumor cell inoculation. The growth of UN‐SSC680^L858R^‐derived tumors was examined every 5–6 days by measuring tumor size using a digital caliper. Tumor volumes were calculated using the formula: *V* = (length × width × width)/2.

For the EVax and IL‐17a/IFN‐*γ* neutralization experiments, wild‐type female A/J mice were purchased from the Jackson Laboratory. 5–6 week‐old mice were grouped according to similar bodyweights into the following four groups: 1) vehicle/adjuvant control mice that received PBS i.p. injections and CFA/IFA injections; 2) vaccinated mice that received PBS i.p. injections and two EGFR peptides plus adjuvant injections; 3) vaccinated mice that received anti‐17a i.p. injections and two EGFR peptides plus adjuvant injections; 4) vaccinated mice that received anti‐IFN‐*γ* i.p. injections and two EGFR peptides plus adjuvant injections. Anti‐IL‐17A (clone‐17F3, 500 µg, BioXcell, BE0173) and anti‐IFN‐*γ* (clone XMG1.2, 1 mg, BioXcell, BE0055) were i.p. injected every Monday and Friday until the end of the experiments. UN‐SSC680^L858R^ tumor cell inoculation and EVax administration were the same as in the earlier anti‐tumor efficacy experiments.

### H&E and IHC Staining

Immediately after euthanasia, lungs from the various treatment groups were inflated, harvested, formalin‐fixed overnight, and then stored in 70% ethanol and processed (Sakura Tissue Tek VIP5) for paraffin embedding. After paraffin embedding, samples were sectioned at 4 µm (Microm HMS355S) onto poly‐l‐lysine‐coated slides and air‐dried at 45 °C overnight prior to H&E and IHC staining.

For H&E stain, five slides were cut per lung with 100 µm spacing between slides. H&E staining slides were scanned using the NanoZoomer HT slide scanner (Hamamatsu, Photonics) and were further analyzed using NanoZoomer software. Tumor burden was quantified by Color Threshold module plus the Measure Image Area module in ImageJ after color deconvolution and interpreted as positive tumor area divided by total lung area (in percentage) per slide. Lung tumor area percentage for each mouse was calculated as the average of this parameter in all five slides of the same mouse.

Fixed lung tissues were further processed with IHC staining with anti‐CD4 (Abcam, cat.: AB183685, 1:50 dilution) or anti‐CD8 (Cell Signaling, cat.: 98941, 1:400 dilution) primary antibodies. Slides were applied with enzyme conjugate secondary anti‐mouse antibody, followed by adding DAB substrate‐chromogen mixture. IHC slides were scanned with the Aperio AT2 digital whole slide scanner (Leica) and were further analyzed using Aperio Imagescope software. The number of positive cells in the field was expressed as % of CD8^+^ cells per mm^2^ tumor area. Data were shown as the mean ± SEM.

### In Vitro T Cells Activation and Th1 Cells Differentiation Studies

To generate Th1 cells, naïve CD4^+^ T cells were first negatively isolated from the spleens of the A/J mice using separation cocktail (Miltenyi, cat: 130‐104‐454) and LS column (Miltenyi). One million cells/mL of naïve CD4^+^ T cells in 6‐well plate were activated with plate‐bound anti‐CD3 (1 µg mL^−1^), soluble anti‐CD28 (2 µg mL^−1^), and mouse Th1 differentiation supplement (1:100 dilution) in the presence of vehicle or DON (1 µm) for 3 days. From day 3, cells were moved to non‐coated 10‐cm dishes, expanded and differentiated with mouse Th1 differentiation supplement (1:100 dilution) for 2 additional days with continued vehicle or DON. On day 6, cells were harvested for FACS analysis. To generate activated CD8^+^ T cells, naïve CD8^+^ T cells were first negatively isolated from the spleens of the A/J mice using separation cocktail (Miltenyi, cat: 130‐104‐075) and LS column (Miltenyi). One million cells/mL of naïve CD8^+^ T cells in 6‐well plate were activated with plate‐bound anti‐CD3 (1 µg mL^−1^), soluble anti‐CD28 (2 µg mL^−1^), and mouse IL‐2 (50 U mL^−1^) in the presence of vehicle or DON (1 µm) for 3 days. On day 3, cells were harvested for seahorse analysis. CD8^+^ cells were moved to the non‐coated 10 cm dishes for expansion in T cells media supplement with mIL‐2 (50 U mL^−1^) for 2 additional days with continued vehicle or DON for FACS analysis of memory markers. For FACS analysis of activation markers, after the initial 2 days of activation, cells were moved to non‐coated plates for 4 additional days, expanded on day 2 and day 4 with mouse IL‐2 (50 U mL^−1^) and continued vehicle or DON exposure, then cells were subjected to flow analysis. Cells were washed and stained as in the flow cytometry method section. For Seahorse metabolic flux analyses, isolated CD4^+^ T cells were activated for 3 days and polarized into Th1 cells by day 5 (as described above); CD8^+^ T cells were activated for 3 days and were used for seahorse assay. T cells were washed and plated on Seahorse metabolic flux analysis plates, 1.5 × 10^5^ cells per well in a 96‐well plate and analyzed as described below.

### Seahorse Assay

Th1 cells and CD8^+^ T cells were activated and cultured with either vehicle or DON (1 µm) as mentioned above. For lung tumor cells, 1 × 10^6^ cells of UN‐SCC680^L858R^ cells were plated on 10 cm dishes in puromycin (1 µg mL^−1^) supplemented RPMI‐based media for 24 h, after which media was changed to media containing vehicle or DON (1 µm) for the next 24 h. Cells were washed and trypsinized, counted and plated for seahorse analysis (1.5 × 10^5^ cells/well). For oxygen consumption rates (OCR) determination, cells were incubated in XF Base Medium (Seahorse Bioscience) supplemented with 2 mmol L^−1^ glutamine, 10 mmol L^−1^ glucose, and 1 mmol L^−1^ pyruvate for 1 h in a non‐CO_2_ incubator. Cells were analyzed under stressed conditions and in response to oligomycin (1.5 µm), carbonyl cyanide 4‐(trifluoromethoxy)phenylhydrazone (2 µm), and rotenone and antimycin A (1 µm) using a Seahorse XF96 extracellular flux analyzer (Agilent Technologies). Basal OCR reports were generated by Wave Desktop software (Agilent Technologies).

### Flow Cytometry

For immune profiling, the middle lung lobe from each mouse was harvested at the end of the study, minced into 1–2 mm pieces, digested at 37 °C for 30 min with mouse tumor dissociation buffer (Miltenyi Biotec, CA), and passed through a 40 µm nylon mesh to generate single‐cell suspensions. Isolated cells were first stained with viability and cell surface markers. Violet fluorescent reactive dye (Cat. No. MP34955, Invitrogen) was used to identify live cells from dead cells. Surface marker staining antibodies included: BV786 anti‐CD45 (Clone: 30‐F11), PerCP‐Cy5.5. anti‐CD45 (Clone: 30‐F11), PE anti‐CD3 (Clone:17A2), FITC anti‐CD4 (Clone: GK1.5), BUV396 anti‐CD8a (Clone: 53‐6.7), BV510 anti‐CD8a (53‐6.7), FITC anti‐CD11b (Clone: M1/70), APC/Fire750 anti‐F4/80 (Clone: BM8), BUV396 anti‐Ly6G (Clone: 1A8), PE/Cy7 anti‐Ly6C (Clone: HK1.4), APC anti‐CD107a (Clone: 1D4B), APC/Fire750 anti‐CD25 (Clone: PC61), PE/Cy7 anti‐CD44 (Clone: 1M7), Alexa flour 700 anti‐CD44 (Clone: 1M7), APC anti‐CD69 (Clone: H1.2F3), PE‐CF594 anti‐CD69 (Clone: H1.2F3), BV605 anti‐CD62L (Clone: MEL‐14), PE‐pSTAT5 (Cat: 14603s, cell signaling Technology), and PE‐c‐MYC (Clone: Cat: 35876s, cell signaling Technology). For transcription factor staining, cells were first stained with surface markers, then fixed with 5% paraformaldehyde, and permeabilized (FoxP3/Transcription Factor Staining Buffer Set, eBioscience, 00‐5523‐00) and stained with APC anti‐FoxP3 (Clone: FJK‐16s). For CD107a degranulation assay, cells were stimulated overnight in an anti‐CD107a antibody added RPMI media containing EGFR peptides (10 µg mL^−1^), 10% FBS, 2 mM L‐glutamine, 50 µM 2‐mercaptoethanol, and 1% penicillin‐streptomycin and HEPES. 1× monensin (eBioscience, 00‐4505‐51) and 1× Brefeldin A (eBioscience, 00‐4506‐51) were added during the last 4 h of stimulation. After stimulation, cells were first surface stained with antibodies, fixed with 5% paraformaldehyde, washed, and then analyzed by flow cytometry. Cells were incubated in media without peptides but containing monensin and Brefeldin A served as non‐stimulated control. For p‐STAT5 and c‐MYC staining, cells were first stained with surface markers, fixed with 5% paraformaldehyde, and permeabilized using pre‐chilled (−20 °C) pure methanol. Then, cells were washed and stained with PE‐conjugated anti‐p‐STAT5 and anti‐c‐MYC antibodies for 20 min at room temperature. To analyze myeloid‐derived cells, cells were additionally incubated with anti‐Mo CD16/CD32 (Invitrogen, 14‐0161‐82) to block background staining. Cells were acquired using the LSR Fortessa X‐20 flow cytometer (Becton Dickinson). Data were analyzed using FlowJo software (Version 10.4). Only live cells were analyzed, and doublets were excluded.

### scRNA‐Seq

For scRNA‐seq, right middle lung lobes were harvested from mice in each treatment group at the end of the study, then minced and digested at 37 °C for 30 min with mouse tumor dissociation buffer (Miltenyi Biotec, CA) to generate single‐cell suspensions per the manufacturer's instructions. TILs were directly stained with violet viability dye, APC anti‐CD45, and CD45^+^ leukocytes were FACS sorted out. FACS‐sorted CD45^+^ leukocytes were then spun down at 300 × *g* for 5 min, counted manually with Neubauer Chamber. Approximately 2.0 × 10^4^ cells were loaded onto the 10x Chromium Controller per the manufacturer's instruction, resulting in a recovery of about 1 × 10^4^ cells. The scRNA‐seq libraries were generated by Chromium Single Cell 3' v3 Reagent Kits (10x Genomics) and sequenced using NextSeq 500/550 High Output Kits v2 (150 cycles) (Illumina) according to the manufacturer's protocol. There were two replicates for each experimental group.

### scRNA‐Seq Data Analysis

Raw sequencing data were de‐multiplexed and converted to gene‐barcode matrices using the Cell Ranger (version 2.2.0) mkfastq and count functions, respectively (10x Genomics). The mouse reference genome mm10 was used for alignment. Data were further analyzed in R (version 3.4.0) using Seurat (version 3). The number of genes detected per cell, number of unique molecular identifiers (UMIs), and the percent mitochondrial genes were plotted, and outliers were removed to filter out doublets and dead cells. Raw UMI counts were normalized and log‐transformed. Integrated analysis was then performed to identify shared cell clusters that were present across different datasets. Principal component analysis was performed using variable genes, and the top 20 most statistically significant principal components were used for t‐SNE or UMAP analysis. For the pathway activity analyses, different immune cell populations were identified by using the Seurat program. When scoring cells for the expression of the above‐known gene signatures, the AddModuleScore function was used in Seurat. The comparison of the signature scores of the cells of each immune population across different groups was performed using the functions implemented in the rstatix R package (https://rpkgs.datanovia.com/rstatix/).

### Bulk RNA‐Seq Data Analysis

To validate the pathway activity analysis results from scRNA‐seq, bulk RNA‐seq data were downloaded from three previous studies. The first one performed RNA‐sequencing on CD4^+^ Th1 cells from either vehicle or CB839 that was a GLS inhibitor (GSE112244).^[^ ^18^ ^]^ The second study performed RNA‐sequencing on sorted CD8^+^ TILs from vehicle or JHU083‐treated MC38 tumor‐bearing mice (GSE120345).^[^ ^6^ ^]^ The third study conducted RNA‐sequencing on sorted TAM cells from 4T1 tumor (murine breast cancer) bearing mice with or without JHU083 treatment (GSE119733).^[^ ^10^ ^]^ The DESeq2^[^ ^38^ ^]^ and fgsea (https://bioconductor.org/packages/fgsea/) R packages were used to conduct the differential gene expression analysis and subsequent pathway activity analyses. The raw RNAseq count data available from the above three studies were downloaded from the GEO database (https://www‐ncbi‐nlm‐nih‐gov.ezproxy.u‐pec.fr/gds). The software tximport^[^ ^39^ ^]^ was used to import the downloaded raw data and summarize abundance estimates for gene‐level analysis with DESeq2. The results from DESeq2 were further analyzed by fgsea for validating the pathway activity changes detected in the scRNA‐seq analysis.

### Statistical Analysis

In terms of sample size, six to nine mice per group were used for in vivo studies. The GraphPad Prism 8.0 was used for statistical analysis. The results were presented as mean ± SE. Student *t*‐test and one‐Way ANOVA were employed for statistical comparison. Statistical significance was determined with 95% (*, *p* < 0.05), 99% (**, *p* < 0.01), 99.9% (***, *p* < 0.001), and 99.99% (****, *p* < 0.0001) confidence intervals.

## Conflict of Interest

Author B.S.S. is an inventor on patent applications filed by Johns Hopkins Technology Ventures covering novel glutamine antagonists, including JHU083, and their utility which have been licensed to Draren Pharmaceuticals. B.S.S. is also a co‐founder of and a consultant for Dracen Pharmaceutials. This arrangement has been reviewed and approved by Johns Hopkins University in accordance with their respective conflict of interest policies. Other authors declare no conflict of interest.

## Supporting information

Supporting Information

## Data Availability

The data that support the findings of this study are available from the corresponding author upon reasonable request.
