# Supplementary material for: Targeting Glutamine Metabolism to Enhance Immunoprevention of EGFR‐Driven Lung Cancer
Source: Adv Sci (Weinh). 2022 Jul 21;9(26):2105885. doi: 10.1002/advs.202105885 (PMC9475521; doi:10.1002/advs.202105885)
Supplement: Supplementary file 1 — Supporting Information [file ADVS-9-2105885-s001.pdf]

ADVANCED SCIENCE
Open Access

Supporting Information

for *Adv. Sci.*, DOI 10.1002/advs.202105885

Targeting Glutamine Metabolism to Enhance Immunoprevention of EGFR-Driven Lung Cancer

*Mofei Huang, Donghai Xiong, Jing Pan, Qi Zhang, Shizuko Sei, Robert H. Shoemaker, Ronald A. Lubet, Luis M. Montuenga, Yian Wang, Barbara S. Slusher and Ming You**

A

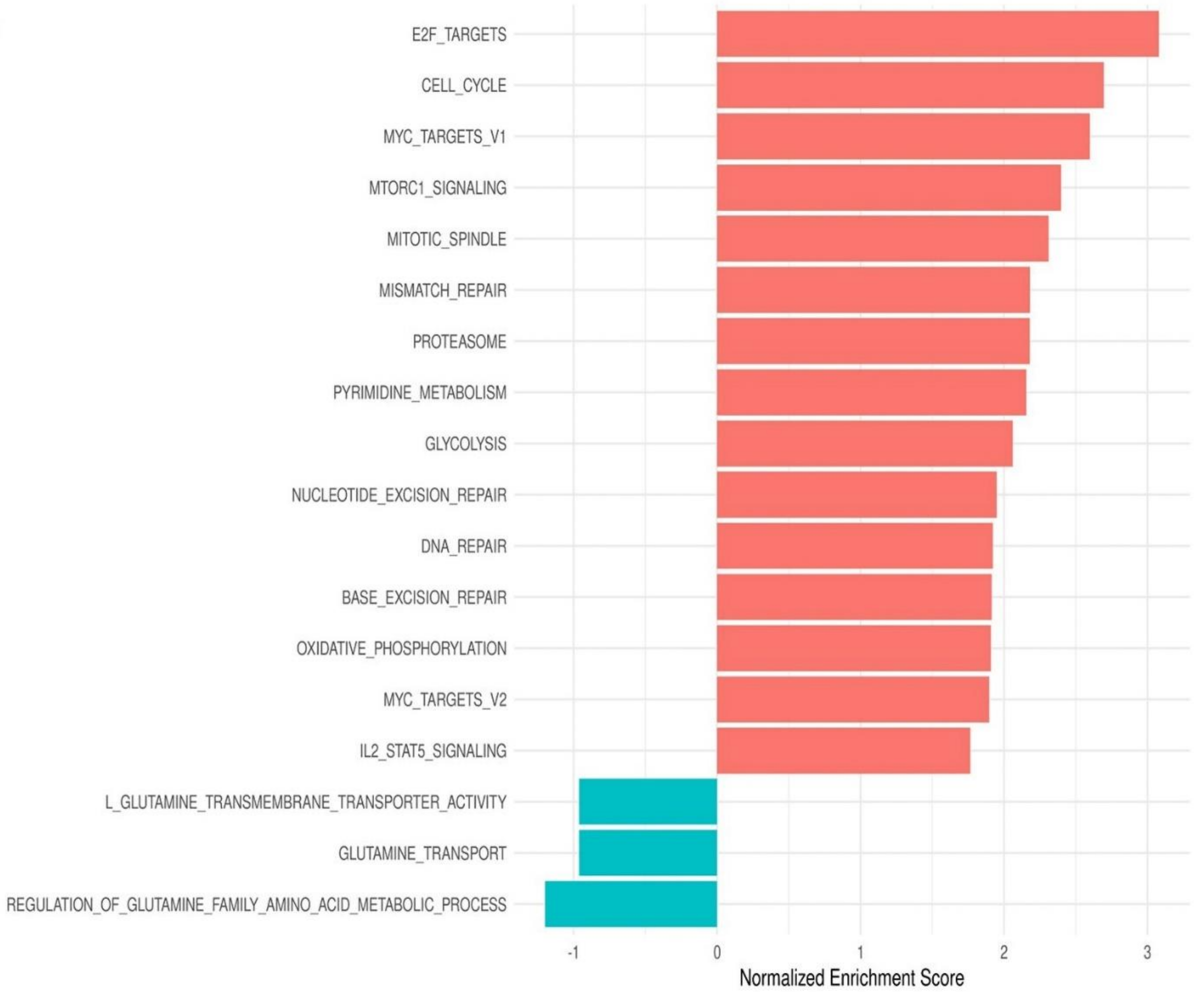
E2F_TARGETS
CELL_CYCLE
MYC_TARGETS_V1
MTORC1_SIGNALING
MITOTIC_SPINDLE
MISMATCH_REPAIR
PROTEASOME
PYRIMIDINE_METABOLISM
GLYCOLYSIS
NUCLEOTIDE_EXCISION_REPAIR
DNA_REPAIR
BASE_EXCISION_REPAIR
OXIDATIVE_PHOSPHORYLATION
MYC_TARGETS_V2
IL2_STAT5_SIGNALING
L_GLUTAMINE_TRANSMEMBRANE_TRANSPORTER_ACTIVITY
GLUTAMINE_TRANSPORT
REGULATION_OF_GLUTAMINE_FAMILY_AMINO_ACID_METABOLIC_PROCESS
-1
0
1
2
3
Normalized Enrichment Score

B

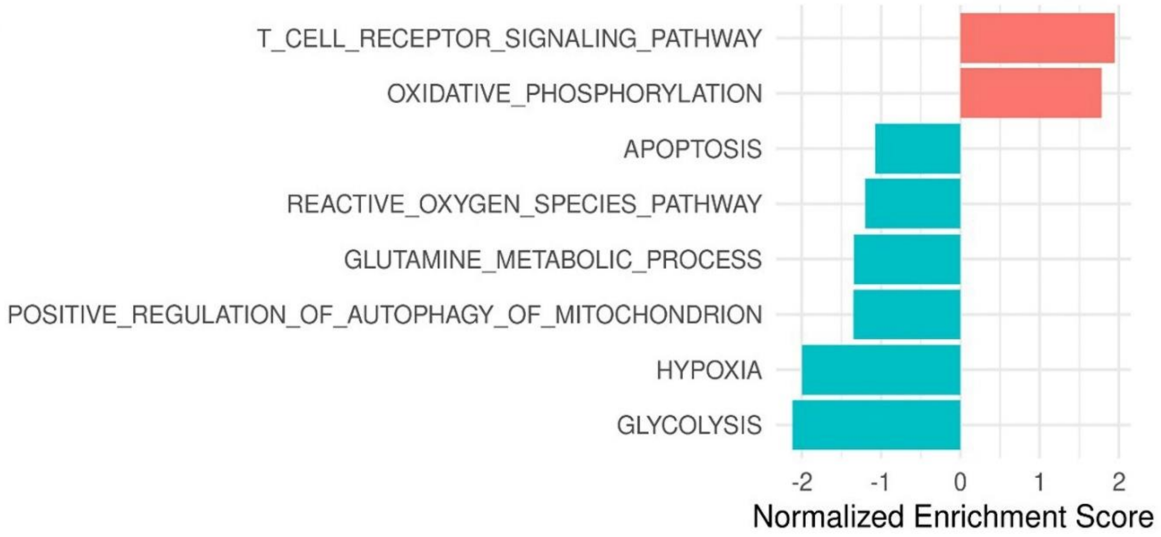
T_CELL_RECEPTOR_SIGNALING_PATHWAY
OXIDATIVE_PHOSPHORYLATION
APOPTOSIS
REACTIVE_OXYGEN_SPECIES_PATHWAY
GLUTAMINE_METABOLIC_PROCESS
POSITIVE_REGULATION_OF_AUTOPHAGY_OF_MITOCHONDRION
HYPOXIA
GLYCOLYSIS
-2
-1
0
1
2
Normalized Enrichment Score

C

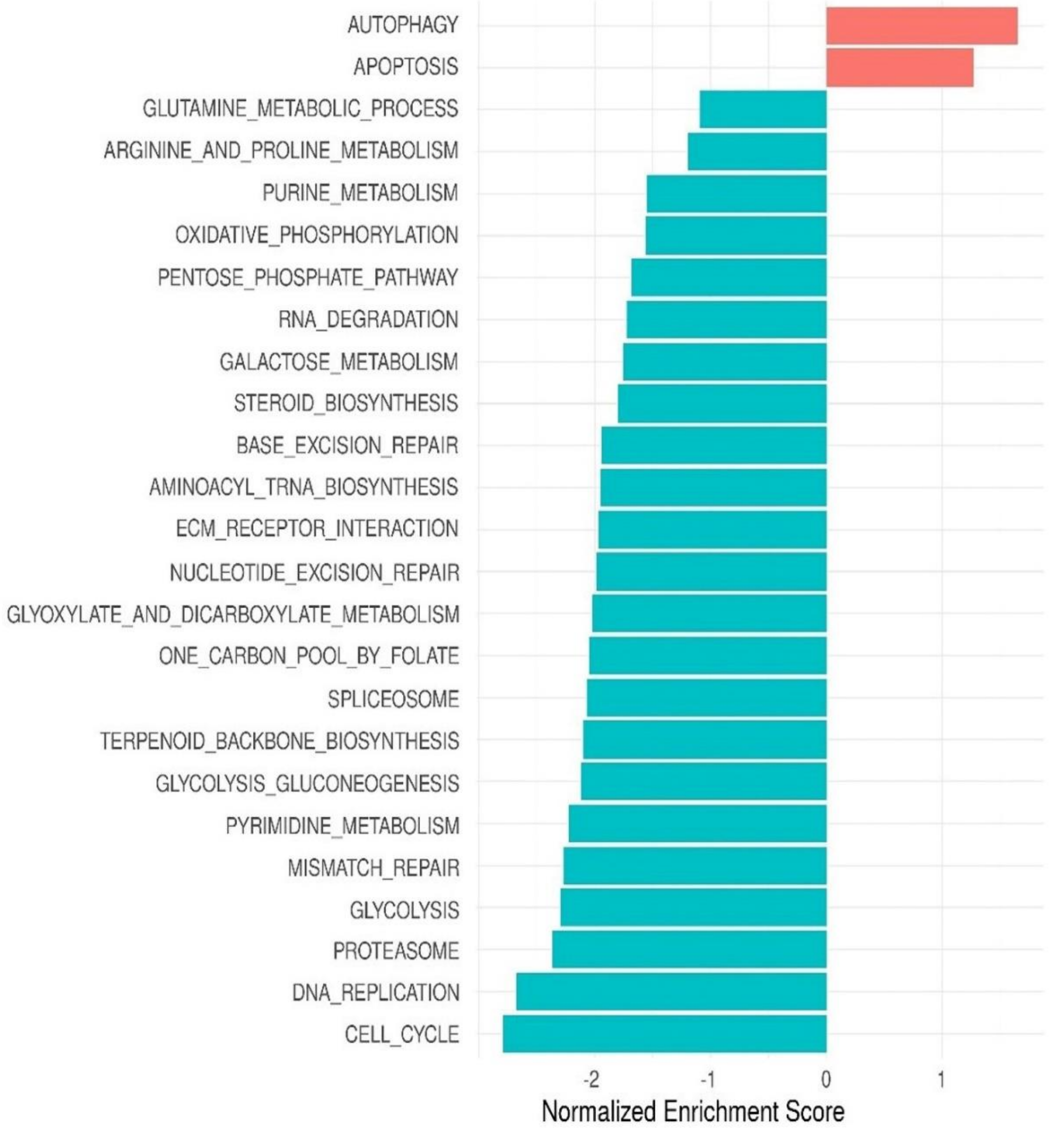
AUTOPHAGY
APOPTOSIS
GLUTAMINE_METABOLIC_PROCESS
ARGININE_AND_PROLINE_METABOLISM
PURINE_METABOLISM
OXIDATIVE_PHOSPHORYLATION
PENTOSE_PHOSPHATE_PATHWAY
RNA_DEGRADATION
GALACTOSE_METABOLISM
STEROID_BIOSYNTHESIS
BASE_EXCISION_REPAIR
AMINOACYL_TRNA_BIOSYNTHESIS
ECM_RECEPTOR_INTERACTION
NUCLEOTIDE_EXCISION_REPAIR
GLYOXYLATE_AND_DICARBOXYLATE_METABOLISM
ONE_CARBON_POOL_BY_FOLATE
SPLICEOSOME
TERPENOID_BACKBONE_BIOSYNTHESIS
GLYCOLYSIS_GLUCONEOGENESIS
PYRIMIDINE_METABOLISM
MISMATCH_REPAIR
GLYCOLYSIS
PROTEASOME
DNA_REPLICATION
CELL_CYCLE
-2
-1
0
1
Normalized Enrichment Score

Supplementary Figure 1. Pathway activity analysis of the previous two RNA-seq datasets comparing the JHU083 treatment group to the untreated control group. (A) Pathway activity changes in Th1 cells after CB839 treatment; (B) Pathway activity changes in Cd8+ TILs under the JHU083 treatment; (C) Pathway activity changes in tumor-associated macrophage (TAM) cells under the JHU083 treatment.
